# Infection of human sweat glands by SARS-CoV-2

**DOI:** 10.1038/s41421-020-00229-y

**Published:** 2020-11-13

**Authors:** Jia Liu, Yufeng Li, Liang Liu, Xudong Hu, Xi Wang, Hengrui Hu, Zhihong Hu, Yiwu Zhou, Manli Wang

**Affiliations:** 1 State Key Laboratory of Virology, Wuhan Institute of Virology, Center for Biosafety Mega-Science, Chinese Academy of Sciences, Wuhan, Hubei 430071 China; 2 Department of Forensic Medicine, Tongji Medical College of Huazhong University of Science and Technology, Wuhan, Hubei 430030 China; 3 Wuhan Jinyin-tan Hospital, Wuhan, Hubei 430023 China

**Keywords:** Cellular imaging, Cell growth

Dear Editor,

Severe acute respiratory syndrome coronavirus 2 (SARS-CoV-2) induces multiorgan dysfunction by rampaging throughout the body^1,2^. As dermatological lesions affect 1%–20% of patients with coronavirus disease 2019 (COVID-19)^3^, the skin may not be exempt. Skin biopsy samples reportedly have low SARS-CoV-2 loads^4,5^; however, it remains unclear whether SARS-CoV-2 directly causes cutaneous manifestations, and if so, what is the cell tropism of the virus in the skin and whether skin contact poses a risk of viral transmission.

To explore these issues, we obtained skin autopsy samples from five patients with COVID-19. Although they had no clinical dermatological manifestations (Supplementary Table S1), microscopy revealed that all patients had lymphocyte infiltration—particularly adjacent to the epidermis and accessory glands in the dermis (Fig. 1a, blue arrows; Supplementary Table S1). Additionally, some patients had mild dermatitis characterized by scattered necrotic cells in the epidermis (Fig. 1a, green arrows). Immunohistochemical analysis demonstrated that the infiltrating lymphocytes included CD3^+^/CD8^+^ T cells and CD68^+^ macrophages (Fig. 1b) but not CD4^+^ T cells, CD19^+^/CD20^+^ B cells or myeloperoxidase (MPO)-positive neutrophils (Supplementary Fig. S1a).

**Fig. 1:**
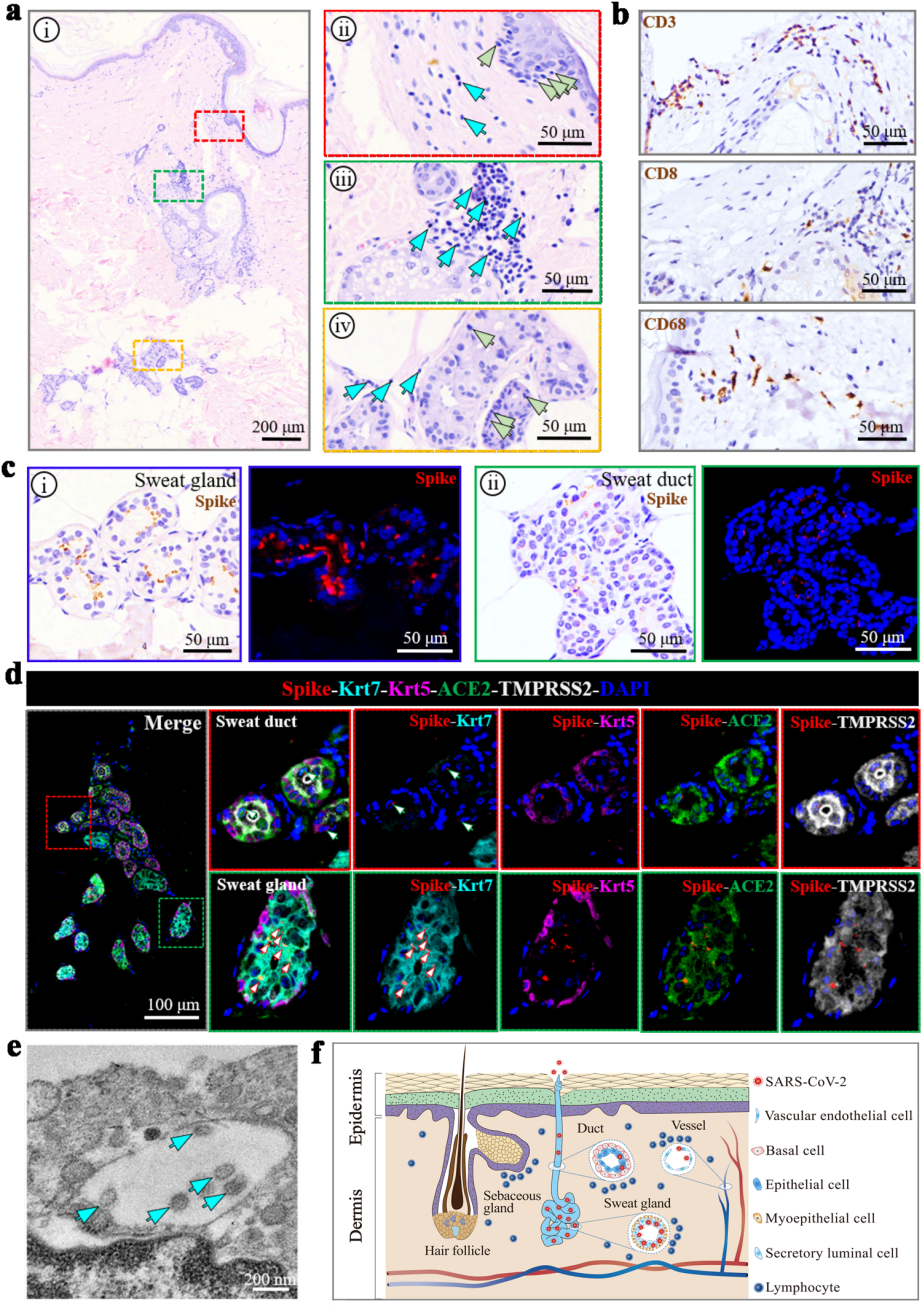
Histopathological and virological analyses of skin tissues from deceased patients with COVID-19. **a** Hematoxylin and eosin staining of skin tissue sections (i–iv). The green and blue arrows indicate necrotic cells in the epidermis and lymphocyte infiltration in the dermis, respectively. **b** Analysis of the immune response in skin tissues. Immunohistochemical staining for inflammatory cell markers, including CD3^+^, CD8^+^ T lymphocytes, and CD68^+^ macrophages. **c** SARS-CoV-2 detection in skin tissue. Immunohistochemical and immunofluorescence analyses of SARS-CoV-2 infection in sweat glands (i) and sweat ducts (ii). **d** Characterization of cells targeted for SARS-CoV-2 infection and correlation between cell tropism and receptor distribution in the skin. Multicolor immunofluorescence staining of SARS-CoV-2 spike protein (red), cell markers of secretory luminal cells (Krt7; cyan) and basal cells (Krt5; magenta), ACE2 receptor (green), and TMPRSS2 coreceptor (gray). The right panels are enlarged images of the respective colored boxes on the left. Arrows indicate representative positive signals for viral spike. **e** Electron microscopy of viral particles (arrows) in the skin cell. **f** Schematic of SARS-CoV-2 infection in the skin.

Immunofluorescence and immunohistochemical analyses detected SARS-CoV-2 spike proteins in three of the five patients (Supplementary Table S1). In these cases, the virus resided primarily in the sweat glands and sweat ducts with apparently higher amounts in the former than in the latter (Fig. 1c); in contrast, the virus was rarely detected in the epidermis or sebaceous glands (Supplementary Fig. S1b).

Sweat glands comprise the inner secretory luminal and outer myoepithelial cell layers, while sweat ducts comprise epithelial and basal cells^6^. To explore the details of SARS-CoV-2 cell tropism, colocalization analysis of viral spike proteins and individual cell markers was performed. In sweat glands, the keratin (Krt) 7^+^ secretory luminal cells were found to be major target cells for SARS-CoV-2 infection, whereas the Krt5^+^ cells/alpha smooth muscle actin (α***-***SAM)^+^ myoepithelial cells were not infected (Supplementary Fig. S2a, the middle panel). In sweat ducts, some Krt5^−^/Krt7^−^ epithelial cells, but not Krt5^+^ basal cells, were infected (Supplementary Fig. S2a, the right panel). Colocalization analysis using a multicolored set of cell markers further confirmed that SARS-CoV-2 primarily targeted Krt7^+^ secretory luminal cells of the sweat glands; additionally it infected the Krt5^−^/Krt7^−^ epithelial cells of the sweat ducts (Fig. 1d, arrows).

Cell surface receptors are a major determinant for the cell tropism of a virus. SARS-CoV-2 exploits the angiotensin-converting enzyme 2 (ACE2) receptors and transmembrane protease serine 2 (TMPRSS2) coreceptors for efficient cell entry^7,8^. To determine the possible relationship between cellular receptors/coreceptors and the cell tropism of SARS-CoV-2, we analyzed the distribution of ACE2 and TMPRSS2 in relation to viral spike proteins in various sweat gland and sweat duct cells. In sweat gland, we found that ACE2 and TMPRSS2 were abundantly expressed in the luminal secretory cells, and viral spike protein distribution corresponded with that of ACE2/TMPRSS2 (Supplementary Fig. S2b, the middle panel). In sweat ducts, the expression levels of TMPRSS2 were higher than those of ACE2, and they appeared to be separately expressed in distinct cells (Supplementary Fig. S2b, the right panel). Multicolor immunofluorescence analysis confirmed the co-expression of ACE2 and TMPRSS2 in Krt7^+^ secretory luminal cells of the sweat glands, and consistently, abundant viral antigens were detected within ACE2^+^/TMPRSS2^+^ cells. By contrast, ACE2 and TMPRSS2 did not show overlapped distribution in sweat ducts (Fig. 1d). Taken together, these results potentially explain the high expression of SARS-CoV-2 spike protein in sweat glands and low expression of SARS-CoV-2 spike protein in sweat ducts. Furthermore, electron microscopy confirmed the presence of viral particles in skin tissues (Fig. 1e).

In addition to sweat glands and ducts, small blood vessels in the skin were also targeted by SARS-CoV-2. Vasculitis characterized by prominent lymphocyte infiltration and swollen vascular endothelial cells was observed (Supplementary Fig. S3a). Immunohistochemical analysis revealed that the infiltrating cells were CD3^+^/CD8^+^ T cells and CD68^+^ macrophages but not CD4^+^ T cells, CD19^+^/CD20^+^ B cells or MPO^+^ neutrophils (Supplementary Fig. S3b); this was similar to the immune cell composition of surrounding skin accessory glands (Fig. 1b; Supplementary Fig. S1a). Accordingly, viral spike proteins were detected in the vascular endothelial cells (CD31^+^) of the dermis (Supplementary Fig. S3c). Vascular endothelial injury and endotheliitis have been reported in some COVID-19 patients, and the virus is known to be capable of directly infecting cultured blood vessel organoids^9,10^. Accordingly, our study supports the potential role of the vascular system in viral pathogenesis and dissemination.

In conclusion, our results suggest that SARS-CoV-2 readily infects sweat gland Krt7^+^ secretory luminal cells coexpressing ACE2 and TMPRSS2. The infection of vascular endothelia suggests that the virus might disseminate to the skin via blood vessels. The immune response of lymphocyte infiltration in skin is likely induced by SARS-CoV-2 infection. A schematic of SARS-CoV-2 infection in the skin is shown in Fig. 1f. As with SARS-CoV^11^, the targeting of sweat glands by SARS-CoV-2 may lead to viral shedding via perspiration. However, we note in our study, that patients with virus-positive sweat glands did not have dermatological symptoms, suggesting that such skin infections might be more common than reported. Therefore, it is important to further assess the potential risk of viral transmission via perspiration and skin contact.

## Supplementary information

Supplementary Information

## Supplementary information

**Supplementary Information** accompanies the paper at (10.1038/s41421-020-00229-y).
