# Supplementary material for: Infection of human sweat glands by SARS-CoV-2
Source: Cell Discov. 2020 Nov 13;6:84. doi: 10.1038/s41421-020-00229-y (PMC7661176; doi:10.1038/s41421-020-00229-y)
Supplement: Supplementary file 1 — Supplementary Information [file 41421_2020_229_MOESM1_ESM.pdf]

Infection of Human Sweat Glands by SARS-CoV-2

Supplementary Appendix: Table of Contents

Page

Materials and Method

Patients, Clinical data, and Workflow 2

Histochemical Staining 2

Imunohistochemical (IHC) Staining 2

Immunofluorescence (IFA) Staining 3

Multiplex Immunofluorescence Assay 3

Transmission Electronic Microscopy (TEM) 4

Supplementary Figures

Figure S1. Histopathological and virological analyses of skin tissues from the deceased patients with COVID-19 5

Figure S2. Characterization of cells targeted for SARS-CoV-2 infection in sweat glands and sweat ducts by dual-color immunofluorescence staining. 6

Figure S3. Histopathological and virological analyses of blood vessel in skin tissues from the deceased patients with COVID-19. 7

Supplementary Tables

Table S1. Clinical data and laboratory tests of the COVID-19 patients 8

Table S2. Information of antibodies used for immohistochemical staining and immunofluorescence assay 9

Materials and Methods

Patients, Clinical data, and Workflow

Skin autopsy samples of five patients with COVID-19 were analyzed. The patients were confirmed SARS-CoV-2 cases admitted to Wuhan Jinyintan Hospital who passed away during February –March, 2020 (basic data are provided in Table S1). The skin tissues of the chest were collected during complete autopsy, which was conducted according to the regulations issued by the National Health Commissions of China and approved by the Ethics Committee of the hospital (permission number: KY-2020-15.01). Before autopsy, written informed consent was also obtained from the patient's family.

Histochemical Staining

Skin tissues were fixed in 10% neutral buffered formalin and processed for formalin fixed paraffin-embedding (FFPE) and sectioning. Serial sections (3 µm-thick) of the FFPE tissue blocks were obtained and stained with hematoxylin and eosin (H&E).

Imunohistochemical (IHC) Staining

After deparaffinization of the sections, heat-mediated antigen retrieval was performed in citrate buffer (pH 6.0) (Sigma-Aldrich) at 95°C for 20-min by microwave. After cooling at room temperature, quenching of endogenous peroxidase was performed with 0.3% hydrogen peroxide for 15 min at room temperature, followed by washing in water for three times, and blocking with phosphate-buffered saline (PBS), pH 7.4 containing 5% bull serum albumin (BSA) overnight at 4 °C. Then the primary antibodies against SARS-CoV-2 spike protein, CD68, CD3, CD4, CD8, CD19, CD20, or MPO were diluted in PBS containing 1% BSA at corresponding concentration

(Table S2) and incubated overnight at 4 °C. After washing with PBS, the sections were incubated with horseradish peroxidase (HRP)-conjugated goat anti-rabbit/mouse IgG antibody (1:500; Proteintech, China) as the second antibody. For chromogenic DAB staining, slides were incubated with peroxidase complex (RBD staining kit, Servicebio), followed by counterstaining with hematoxylin. The diluent solution without primary antibodies was used as negative control. Images were captured using a digital camera under a light microscope (Thermo, Chriscan-SF).

Immunofluorescence (IFA) Staining

Paraffin sections were treated with 0.1% Sudan Black B (Sigma-Aldrich) for 15 min at room temperature to reduce autofluorescence after deparaffinization. Antigen retrievals were performed by microwave, and the sections were blocked overnight with PBS containing 5% BSA at 4 °C. After washing three times, the section was incubated with primary antibodies at corresponding dilution (Table S2) overnight at 4 °C. After washing with PBS for three times, sections were incubated with the secondary antibodies, Alexa Fluor 488/555-conjugated goat anti-rabbit or mouse IgG (Abcam), for 2 h at room temperature before being stained with DAPI (Biyuntian). The sections were cover-slipped using Antifade Mounting Medium (Servicebio) and images were captured by light microscopy (Thermo, Chriscan-SF).

Multiplex Immunofluorescence Assay

Multiplex fluorescence labeling was performed by TSA-dendron-fluorophores (opal 7-color Manual IHC kit, Perkinelmer, NEL811001kt). Briefly, after deparaffinization, antigen retrieval, and quenching of the endogenous peroxidase, the sections were washed with TBST for 3 min, and then incubated with blocking buffer for 10 min at room temperature. Primary antibody was incubated for 1 hour at room temperature, followed by detection using the HRP-conjugated secondary antibody and TSA-dendron-fluorophores. Afterwards, the primary and secondary antibodies were thoroughly removed by heating the section in retrieval buffer using microwave as

mentioned above. In a serial fashion, each antigen, including (SARS-CoV Spike, Krt5, Krt7, ACE2, and TMRPSS2) (Table S2) was labeled by distinct fluorophores. After all the antibodies were detected sequentially, the sections were stained with DAPI (Biyuntian) and cover-slipped using Antifade Mounting Medium (Servicebio) and imaging using Perkinelmer VECTRA3 automatic quantitative analysis system for tissue sections.

Transmission Electronic Microscopy (TEM)

A small fraction (<1 mm^3) of freshly dissected skin tissues were fixed and inactivated in 2.5% (v/v) glutaraldehyde for at least 24 hour at 4 ^oC, further fixed in 1% (w/v) osmic acid for 2 hours at 20 ^oC. Then, tissues were dehydrated and infiltrated. The ultrathin sections (80-100 nm) were obtained and further stained with 2% uranyl acetate and lead citrate. The samples were examined by a HITACHI H-7000FA transmission electron microscopy at an accelerating voltage of 200 kV.

Supplementary Figures

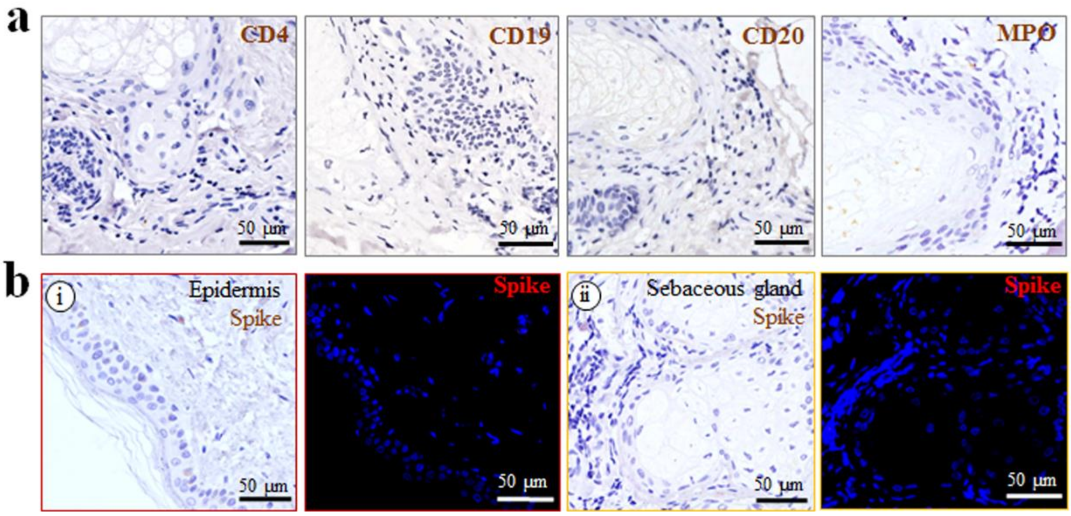

Fig. S1 Histopathological and virological analyses of skin tissues from the deceased patients with COVID-19. a Analysis of immune response in skin tissues. Immunohistochemical staining results for the inflammatory cell markers, including CD4, CD19, CD20, and MPO, were negative. **b** SARS-CoV-2 detection in skin tissues. Immunohistochemical and immunofluorescence analyses showed SARS-CoV-2 rarely infected in the epidermis (i) and sebaceous gland (ii).

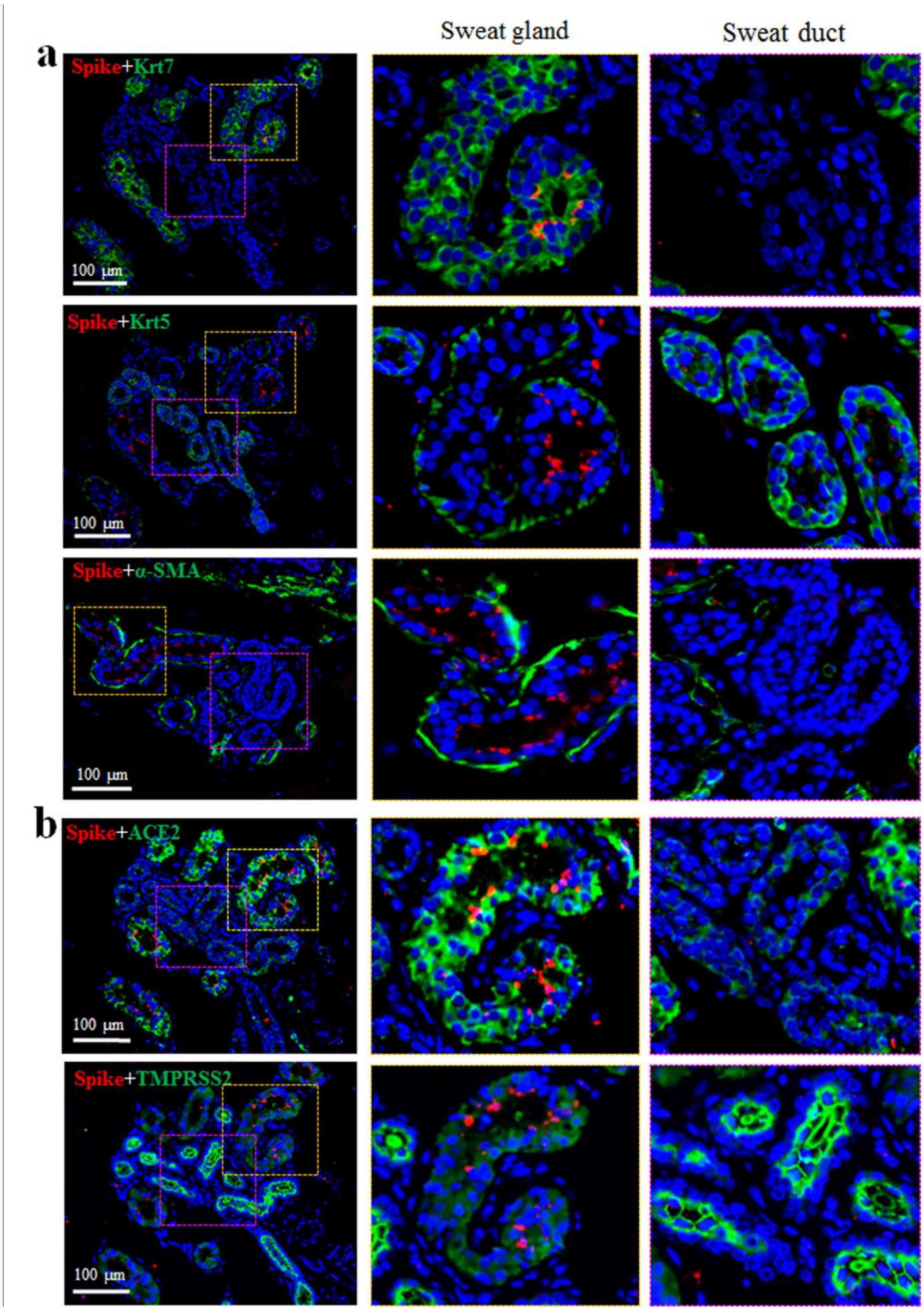

Fig. S2 Characterization of cells targeted for SARS-CoV-2 infection in sweat glands and sweat ducts by dual-color immunofluorescence staining. a Colocalization analyses of SARS-CoV-2 spike protein (red) with Krt7 (for secretory luminal cells), Krt5 (for basal cells), and α-SMA (for myoepithelial cells). Arrows indicated representative signals of viral antigens in sweat ducts. **b** Colocalization analyses of SARS-CoV-2 spike protein (red) with ACE2 or TMPRSS2 coreceptor (green) were performed. The middle and right panels are enlarged images of the respective colored boxes on the left.

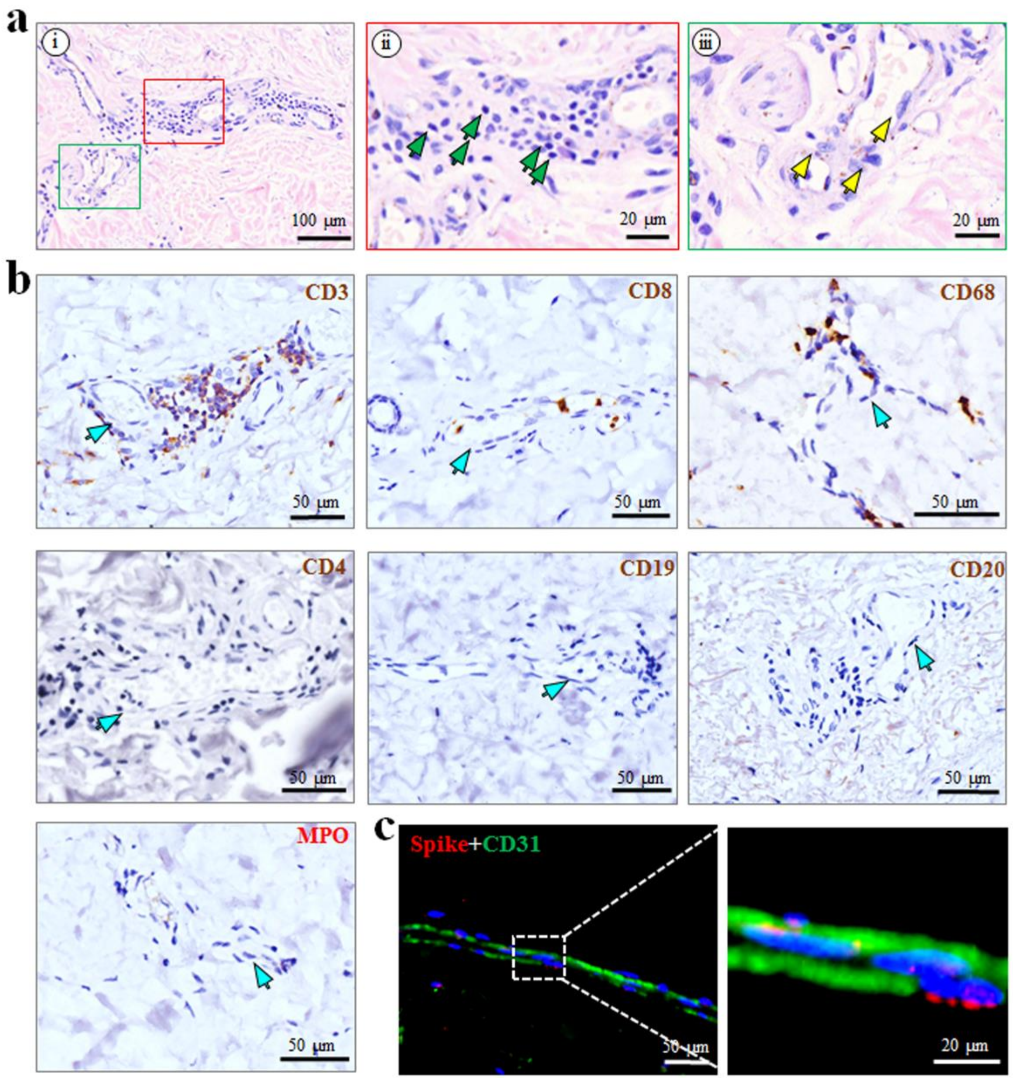

Fig. S3 Histopathological and virological analyses of blood vessel in skin tissues from the deceased patients with COVID-19. a Result of Hematoxylin and eosin staining (i-iii). The green (ii) and yellow (iii) arrows indicate lymphocyte infiltration and swollen vascular endothelial cells around the blood vessel, respectively. **b** Analysis of immune response. Immunofluorescence staining of cells around blood vessels in the skin tissues showed positive for $CD3^+$/$CD8^+$ T lymphocytes, and $CD68^+$ macrophages, and negative for $CD4^+$ T lymphocytes, $CD19^+$/$CD20^+$ B lymphocytes, or MPO^+-neutrophils. The blue arrows indicated blood vessels. **c** SARS-CoV-2 detection in the vascular endothelial cells ($CD31^+$). Dual-color immunofluorescence analysis showed SARS-CoV-2 spike proteins in the vascular endothelial cells. The right figure is the enlarged image of the white box on the left.

Supplementary Tables

Table S1. Clinical data and laboratory tests of the COVID-19 patients

Patients No.	Sex	Age (years)	Death time (days after onset)	Inflammatory infiltration	Virus detection in sweat gland
Case 1	Female	66	28	+	+
Case 2	Female	53	32	+	+
Case 3	Female	86	24	+	-
Case 4	Male	62	31	+	-
Case 5	Male	51	28	+	+

Table S2. Information of antibodies used for immunohistochemical staining and immunofluorescence assay

Primary antibodies	Company	Catalog	Host	Dilutions
Spike	Made in-house	--	Mouse	1:300
CD3	Abcam	ab5690	Rabbit	1:100
CD4	Servicebio	GB13064-1	Mouse	1:100
CD8	Servicebio	GB11068-1	Rabbit	1:200
CD19	Cell signaling tech	#90176	Rabbit	1:400
CD20	Cell signaling tech	#98708	Rabbit	1:200
CD68	Cell signaling tech	#76437	Rabbit	1:400
MPO	Boster	BA0544	Rabbit	1:200
Krt5	Abcam	ab64081	Rabbit	1:100
Krt7	Cell signaling tech	#4465	Rabbit	1:100
CD31	Boster	A01513-3	Rabbit	1:200
ACE2	Abcam	ab15348	Rabbit	1:500
TMRPSS2	Abcam	ab92323	Rabbit	1:500
